# Adverse-Weather Image Restoration Method Based on VMT-Net

**DOI:** 10.3390/jimaging11110376

**Published:** 2025-10-26

**Authors:** Zhongmin Liu, Xuewen Yu, Wenjin Hu

**Affiliations:** 1 School of Electrical Engineering and Information Engineering, Lanzhou University of Technology, Lanzhou 730050, China; 2 School of Mathematics and Computer Science, Northwest Minzu University, Lanzhou 730030, China

**Keywords:** image restoration, adverse weather, Vision-Mamba Transformer, adaptive content guidance

## Abstract

To address global semantic loss, local detail blurring, and spatial–semantic conflict during image restoration under adverse weather conditions, we propose an image restoration network that integrates Mamba with Transformer architectures. We first design a Vision-Mamba–Transformer (VMT) module that combines the long-range dependency modeling of Vision Mamba with the global contextual reasoning of Transformers, facilitating the joint modeling of global structures and local details, thus mitigating information loss and detail blurring during restoration. Second, we introduce an Adaptive Content Guidance (ACG) module that employs dynamic gating and spatial–channel attention to enable effective inter-layer feature fusion, thereby enhancing cross-layer semantic consistency. Finally, we embed the VMT and ACG modules into a U-Net backbone, achieving efficient integration of multi-scale feature modeling and cross-layer fusion, significantly improving reconstruction quality under complex weather conditions. The experimental results show that on Snow100K-S/L, VMT-Net improves PSNR over the baseline by approximately 0.89 dB and 0.36 dB, with SSIM gains of about 0.91% and 0.11%, respectively. On Outdoor-Rain and Raindrop, it performs similarly to the baseline and exhibits superior detail recovery in real-world scenes. Overall, the method demonstrates robustness and strong detail restoration across diverse adverse-weather conditions.

## 1. Introduction

With the rapid advancement of computer vision technology, restoring images degraded by adverse weather has become a critical challenge [1]. During the restoration of images affected by common severe weather conditions (such as rain, fog, and snow), issues such as color distortion, artifacts, and loss of details frequently occur, directly impacting the accuracy and reliability of the restoration process [2]. In recent years, researchers have made progress in single-condition image restoration, including image deraining [3], defogging [4], and desnowing [5]; however, real-world systems often need to handle multiple complex weather conditions simultaneously. Due to the limited generalization of single-task models, they are inadequate for handling compound scenarios in which rain and fog coexist [1,6]. Consequently, designing a generalized restoration framework capable of addressing multiple adverse-weather degradations has emerged as a significant research challenge.

Traditional image restoration methods rely on predefined weather priors to simulate degradation patterns, thus mitigating the impact of adverse weather on image quality [7,8]. However, their performance is sensitive to scene changes, leading to unrealistic restorations and poor generalization. The rapid advances in deep learning have motivated researchers to explore more efficient restoration techniques, advancing the field. Li et al. [6] proposed an end-to-end adverse-weather restoration framework that integrates multiple task-specific encoders into the generator and leverages a neural architecture search mechanism to automatically identify optimal feature-processing paths for different types of weather degradation. Although this approach demonstrates strong adaptability in single-task scenarios, the limited feature sharing among encoders leads to a substantial model size and restricted cross-weather generalization. To address this limitation, Valanarasu et al. [2] introduced a Transformer-based image restoration model with a dedicated encoder–decoder structure, which markedly enhances restoration performance under diverse atmospheric conditions. In addition, they developed an automated data-processing pipeline that improves task adaptability. However, when applied to high-resolution images, the computational cost of Transformers increases quadratically with the input size, and the lack of local inductive bias often results in suboptimal detail recovery. Recent studies have also explored probabilistic denoising diffusion models [9], knowledge distillation [10], mixtures of experts [11], and sample-efficient learning strategies [12] to enhance restoration quality and generalization under complex weather conditions.

Recent advancements in the field include DRSformer, proposed by Chen et al. [13], which integrates sparse attention with a mixed-scale feed-forward network. DRSformer addresses the feature-aggregation interference and multi-scale modeling limitations inherent in conventional Transformers for image deraining. However, it still necessitates task-specific training and tuning, and it lacks the capability to handle diverse weather degradations. To mitigate this issue, Sun et al. [14] introduced Histoformer, an efficient histogram-based Transformer for restoring images degraded by adverse weather conditions. Histoformer incorporates a dynamic-range histogram self-attention mechanism that groups and ranks spatial features by intensity, selectively attending to those within the dynamic range. This mechanism enables joint restoration of rain, fog, snow, and nighttime scenes. However, when processing high-resolution images, the expanded dynamic range leads to a quadratic increase in the computational cost of self-attention with respect to the number of tokens. Moreover, the absence of a local inductive bias limits the Transformer’s sensitivity to high-frequency details, making it challenging to distinguish fine-grained differences between adjacent patches. Furthermore, directly merging multi-level features through naïve skip connections induces spatial-semantic conflicts, leading to artifacts and other instabilities in the restored images.

**Figure 1:**
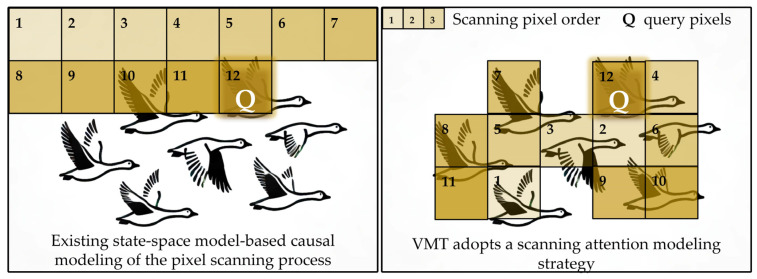
Comparison between causal SSMs and the proposed method. (**Left**): in causal SSMs, a query pixel can access only its historical prefix. (**Right**): our VMT-Net couples DRSA’s non-causal global context with V-Mamba’s single-step linear long-range propagation, enabling simultaneous access to global and local information within a single step.

Zheng et al. [15] proposed a U-shaped Vision Mamba dehazing network (UVM-Net), which leverages the local pattern recognition capability of convolutional neural networks (CNNs) alongside the global contextual modeling power of state-space sequence models (SSMs), thereby improving detection performance under complex weather conditions. However, the unidirectional causal modeling inherent to such approaches is fundamentally incompatible with the non-causal nature of two-dimensional images. As illustrated in Figure 1(left), image restoration—being a typical non-causal task—suffers when an H × W image is flattened into a sequence of length *n* = H × W in raster-scan order, as the t-th pixel can only access information from the preceding t − 1 pixels. This limitation severely restricts the capacity for global context modeling. Furthermore, the local propagation mechanism of SSMs fails to adequately capture long-range dependencies, often resulting in insufficient global consistency or incomplete detail recovery under structurally complex or hybrid degradations, such as snow, fog, or raindrop refraction.

Therefore, the key to adverse-weather image restoration lies in balancing global semantic understanding with local detail recovery. However, existing approaches often face a trade-off: convolutional neural networks (CNNs) are effective at capturing local texture details but struggle with modeling long-range dependencies; Transformers excel at global information modeling but fall short in fine-grained texture restoration and incur prohibitive computational costs; while Mamba achieves efficiency in long-sequence modeling, its representational capacity is limited when directly applied to two-dimensional images.

To address the aforementioned issues, this paper integrates the complementary strengths of Transformers and Mamba by combining non-causal global modeling with single-step linear long-range propagation, as illustrated in Figure 1(right). The Dimensionality Reduction Self-Attention (DRSA) mechanism provides non-causal global context on reduced-dimension tokens, allowing any query position to perceive the entire image in a single step. Meanwhile, Vision-Mamba (V-Mamba) performs coarse-grained contour modeling to capture pixel-level long-range dependencies. These two components interact across positions through the Global Propagation Feedforward Network (GPFN), while the Adaptive Content Guidance Module (ACGM) dynamically weights and fuses features between the encoder and decoder, further enhancing detail reconstruction.

Building on this design, we propose VMT-Net, which embeds DRSA and V-Mamba into a U-Net framework. By coupling them with GPFN-based cross-position propagation and ACGM-based content-gated alignment, the network achieves dynamic feature fusion between the encoder and decoder, significantly improving the restoration of fine details. The main contributions of this paper are as follows:

(1) We propose a Vision-Mamba Transformer (VMT) module that, through a gating mechanism and a global-propagation feed-forward network, effectively fuses the features extracted by V-Mamba and DRSA. This enables synergistic optimization of global context modeling and local structural perception, thereby significantly enhancing the retention of fine details.
(2) We propose an ACGM that combines a dynamic gating mechanism with a spatial-channel attention-guided feature fusion strategy, effectively mitigating the spatial-semantic conflicts induced by direct cross-layer feature weighting. Additionally.
(3) We design a U-Net architecture that integrates both VMT and ACGM, effectively addressing the global semantic loss and local detail blurring commonly encountered in traditional adverse-weather restoration methods due to their limited receptive fields. This architecture significantly improves image consistency and edge sharpness in complex degradation scenarios.

## 2. Related Work

### 2.1. Image Restoration Based on Transformer

Early deep-learning image-restoration research primarily employed convolutional encoder–decoder architectures [13], which excelled at local feature extraction and were straightforward to optimize. However, their limited receptive field hindered the effective modeling of long-range spatial dependencies, thereby restricting the representation of complex structural information. In contrast, Transformer models demonstrate strong global modeling capabilities and achieve superior performance in image restoration tasks, particularly in removing adverse-weather artifacts. Building on this line of research, Liang et al. [16] proposed a restoration network based on the Swin Transformer, where a sliding-window mechanism enhances the correlation between image content and features. However, the fixed-window design restricts cross-window dependencies, often leading to discontinuities at block boundaries and excessive detail smoothing. Subsequently, Zamir et al. [17] introduced the Restormer network, which utilizes multi-scale representations and low-complexity attention to reduce computational burden and mitigate cross-window dependency issues. Nevertheless, in high-resolution and severe degradation scenarios, it still suffers from a global-local imbalance and insufficient cross-layer semantic alignment. To further tackle these challenges, Wang et al. [18] further proposed a network that integrates a triple-attention mechanism to efficiently restore images degraded by multiple severe-weather conditions. By utilizing self-attention to model dependencies across arbitrary spatial positions, Transformer models achieve a global receptive field, significantly enhancing their ability to represent and recover complex scenes, thus driving further advances in image-restoration technology.

Although Transformers demonstrate strong advantages in global semantic modeling, they still suffer from substantial computational complexity and large parameter counts. To mitigate this overhead, practitioners often adopt non-overlapping image patch partitioning. While effective at reducing complexity, this strategy limits fine-grained representation, may introduce boundary artifacts at patch seams, and confines attention to fixed spatial or channel dimensions. Since adverse-weather image restoration requires preserving the natural transition of local textures while maintaining global structural consistency, current methods struggle to achieve a proper balance between these two objectives. To address this challenge, we introduce Mamba, which captures long-range pixel dependencies and enables efficient spatial perception.

### 2.2. Image Restoration Based on Mamba

Mamba is an innovative approach derived from the long-sequence modeling domain; it efficiently processes lengthy sequential data and captures long-range dependencies. Based on the state-space model (SSM) framework, it models relationships among sequence elements within a continuous signal space, thereby significantly enhancing performance on pixel-level global interaction modeling tasks.

Guo et al. [19] were the first to apply Mamba to image restoration, addressing two critical challenges—local pixel forgetting and channel redundancy—with effective solutions, thereby demonstrating the feasibility of state-space models for this task. Building on this, Zou et al. [20] proposed an image denoising method based on Fourier-domain state-space modeling, which improves global degradation awareness to enhance restoration quality; however, interactions between frequency and spatial domains can introduce cross-scale semantic mismatches. Weng et al. [21] further refined the state-space formulation to better capture local details in low-light image enhancement. Nevertheless, the sequential scanning paradigm introduces directional bias and lacks content-adaptive cross-layer alignment between the encoder and decoder. Although Mamba-based models have also shown promising results in tasks such as image dehazing [15] and deblurring [22], they still approximate two-dimensional image restoration as a one-dimensional causal sequence, leading to problems such as limited access to future information and scan-direction bias. Moreover, the absence of content-adaptive cross-layer fusion often causes cross-scale semantic mismatches and color shifts.

## 3. Methodology

### 3.1. Overall Framework

**Figure 2:**
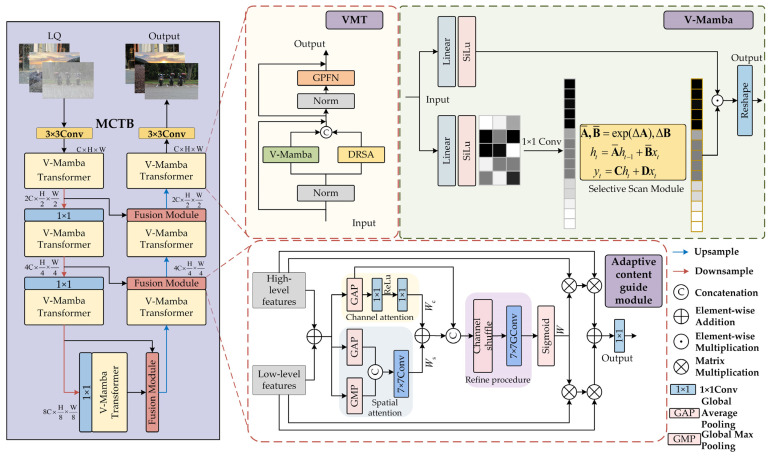
Schematic of the overall network architecture.

We propose a multi-branch Vision-Mamba Transformer (VMT) network, comprising seven hybrid VMT modules and three ACGMs. As the network depth increases, each stage adjusts the channel dimensions using 1 × 1 convolutions and aligns feature-map resolutions through a combination of upsampling and downsampling operations. The overall architecture is depicted in Figure 2.

Given a low-quality input image $I^{lq}\in {\mathbb{R}}^{3\times H\times W}$, a 3 × 3 convolution is first applied to embed image patches and generate the initial feature map. Within the encoder–decoder framework, VMT modules are subse $I^{hq}\in {\mathbb{R}}^{3\times H\times W}$ quently deployed in a layer-wise manner, where each layer incorporates 3 × 3 max pooling and bilinear interpolation-based upsampling to extract multi-scale features. The ACGM utilizes a spatial–channel attention mechanism to adaptively fuse low-level features *F*_low_ with the corresponding high-level decoder output *F*_high_, thereby establishing semantic associations across layers and effectively mitigating degradation of deep features. Finally, a 3 × 3 convolution is applied for pixel-level reconstruction, yielding the restored high-quality image.

### 3.2. Vision-Mamba Transformer Module

In adverse-weather image restoration, most mainstream methods are Transformer-based. However, their limited capacity to represent fine-grained details, combined with the high computational cost of global self-attention, has become a critical bottleneck. To address these limitations, we propose the Vision-Mamba Transformer (VMT) module, which integrates the complementary strengths of Transformer and Mamba to jointly optimize efficiency, global context modeling, and local detail preservation.

The overall network architecture is depicted in 0 The input features are first normalized and then divided into two parallel branches: one branch is processed by the DRSA module for global semantic dependency modeling, while the other passes through the Vision-Mamba (V-Mamba) module for multi-scale feature extraction. The outputs of both branches are concatenated along the channel dimension, followed by another LayerNorm operation, and subsequently fed into a feed-forward network. Within this network, a gated global propagation sub-module facilitates cross-position global information exchange. Finally, the context-enhanced features are added to the residual connection and forwarded to the next stage of the network.

The DRSA mechanism preserves global context modeling while substantially reducing the computational overhead of traditional self-attention. The introduction of the V-Mamba module enhances local feature representation and improves long-range dependency modeling. The GPFN incorporates a gated propagation layer that processes features independently at each global and local position, thereby reinforcing long-range semantic consistency.

#### 3.2.1. Dimensionality Reduction Self-Attention

**Figure 3:**
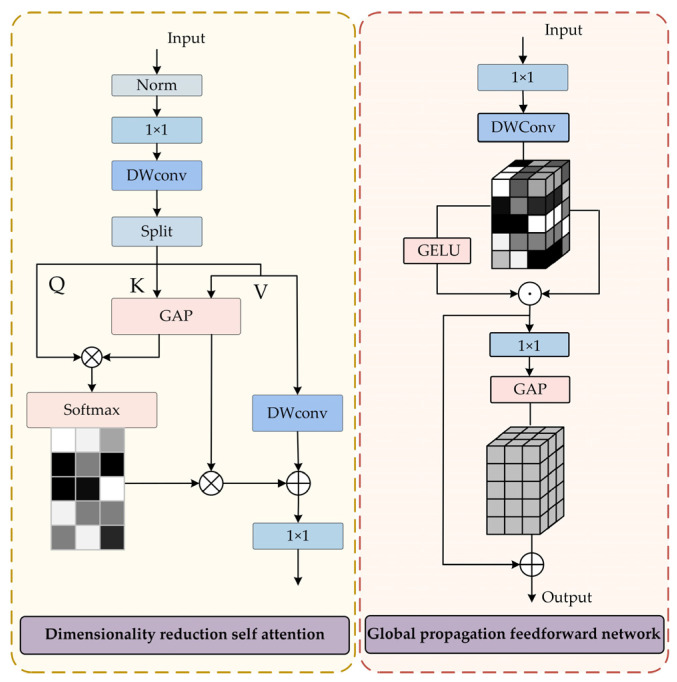
Network architecture of Dimensionality Reduction Self-Attention and Global Propagation Feedforward Network.

In order to effectively balance global semantic modeling and local detail representation in image restoration tasks, we propose a Dimensionality Reduction Self-Attention mechanism, as illustrated in Figure 3. First, the input feature *F* is normalized by applying Layer Normalization, and local features are then extracted using a 1 × 1 convolution followed by a 3 × 3 depth wise separable convolution (DConv_3×3_). This process is formally defined in Equation (1):

(1) $$F^{\prime }={\mathrm{DConv}}_{3\times 3}({\mathrm{Conv}}_{1\times 1}(\mathrm{LayerNorm}(F)))$$

Next, the locally enhanced intermediate features are split along the channel dimension into the query **Q**, key **K**, and value **V** components. In order to reduce the quadratic computational complexity of the attention mechanism, average pooling (AvgPool) is performed on both **K** and **V**, as defined in Equation (2):

(2) $$\begin{array}{l} K^{\prime }=\mathrm{AvgPool}(K)\\ V^{\prime }=\mathrm{AvgPool}(V) \end{array}$$

Subsequently, multi-head attention is employed to compute the global attention weight A, in which the similarity between **Q** and **K** is converted into attention weights through the application of the softmax activation function, such that the weights assigned to all keys for each query sum to one, as formulated in Equation (3):

(3) $$A=\mathrm{softmax}(\frac{QK^{T}}{\sqrt{d_{k}}})$$

where d*_k_* denotes the dimensionality of the key/query in each attention head, $A\in {\mathbb{R}}^{N_{q}\times N_{k}}$. The attention matrix A is used to compute a weighted sum over the linearly projected value vector **V′**, resulting in the long-range dependency representation A**V′**. In order to enhance local detail, the Local Enhancement (LE) module [23] is incorporated by applying a 3 × 3 depthwise separable convolution to the original value feature **V**. This operation captures local context and refines texture information, as defined in Equation (4):

(4) $$V_{\mathrm{LE}}={\mathrm{DWcon}\nu }_{3\times 3}(V)$$

In the final step, the output is generated through residual fusion, which yields the final feature representation, as expressed in Equation (5):

(5) $$F_{d}=V_{\mathrm{LE}}+AV^{\prime }$$

The dual-path strategy of global modeling and local compensation effectively mitigates potential information loss introduced during dimensionality reduction. By capturing holistic structural cues through global modeling and incorporating local convolutions to reinforce fine-grained details, the restored images exhibit sharper edges and more continuous textures that closely resemble the ground truth.

#### 3.2.2. Vision-Mamba Module

To address the limitations of conventional Transformers in terms of modeling long-range dependencies and computational efficiency, a Vision-Mamba (V-Mamba) module is integrated into the Transformer framework, thereby enhancing the network’s ability to capture long-range pixel dependencies. The overall architecture is illustrated in Figure 2.

V-Mamba is a selective state-space model that utilizes a gated selection mechanism to propagate or suppress information based on the current state, thereby enhancing content-reasoning performance. Formally, Mamba captures token interactions through discrete state-space equations, which results in linear computational complexity for long-sequence modeling. The module consists of three key components: feature serialization, dual-path feature modulation, and feature fusion. These are designed to maintain low computational cost while effectively capturing long-range dependencies in images.

(1) Feature serialization

Given an input feature map $F\in {\mathbb{R}}^{B\times C\times H\times W}$, the spatial dimensions are flattened to produce a serialized representation $F^{\prime }\in {\mathbb{R}}^{B\times C\times L}$, where *L = H × W* represents the total number of spatial positions. This serialization step reduces the computational complexity from *O*(*L*^2^) in conventional Transformers to *O*(*L*).

(2) Dual-path feature modulation

Along the primary branch, deep feature transformation is carried out through four sequential operations to capture multi-scale dependencies: (i) channel projection via a Linear layer to reduce redundancy; (ii) application of a SiLU activation to introduce learnable non-linearity; (iii) a 1-D convolution with kernel size k = 3 for local context modeling; and (iv) a State Space Model (SSM) layer, implemented through a discretized state-update equation, to capture long-range dependencies and aggregate global information. The overall procedure is defined in Equation (6):

(6) $$F_{\mathrm{mix}}=\mathrm{SSM}(\mathrm{Conv}1D_{k=3}(\mathrm{SiLU}(\mathrm{Linear}(F^{\prime })))$$

In the gating path, a content-aware weighting mechanism is applied. A Linear layer is first used to transform the features, followed by a SiLU activation that adaptively modulates the weights in accordance with the spatial semantics. This process is formally defined in Equation (7):

(7) $$G=\mathrm{SiLU}(\mathrm{Linear}(F^{\prime }))$$

(3) Feature fusion

The dual-path features are fused via the Hadamard product, followed by spatial reshaping to recover the original two-dimensional spatial layout. This process is defined in Equation (8):

(8) $$F_{m}=\mathrm{Reshape}(F_{\mathrm{mix}}⊙G)\in {\mathbb{R}}^{B\times C\times H\times W}$$

where ⊙ denotes the Hadamard product, and the final output *F*_m_ preserves the original feature map resolution in terms of spatial dimensions.

#### 3.2.3. Global Propagation Feedforward Network

The traditional Gated-Dconv Feedforward Network (GDFN) [17] primarily conducts channel mixing through fully connected layers, which restricts its capacity to model global semantic correlations across spatial dimensions. To address this limitation, a GPFN is proposed, which incorporates an implicit mechanism for global feature injection to propagate global context into local regions, thereby enhancing the model’s ability to capture long-range dependencies. GPFN processes input feature representations from both the multi-head attention mechanism and the Mamba module in two successive stages:

(1) Multi-scale Feature Decoupling

The input feature ***X*** is first passed through a 1 × 1 convolution followed by a 3 × 3 depthwise convolution to expand the channel dimensions, and then split into two groups of deep features. These are processed using the GELU activation function and an element-wise multiplication for gated weighting, producing the enhanced local feature *F*_1_. This process is described in Equation (9):

(9) $$\begin{array}{l} X_{1},X_{2}={\mathrm{DWConv}}_{3\times 3}({\mathrm{Conv}}_{1\times 1}(X))\\ F_{1}={\mathrm{Conv}}_{1\times 1}(\mathrm{GELU}(X_{1}⊙X_{2})) \end{array}$$

(2) Implicit global propagation

A 1 × 1 convolution is initially applied to reduce the channel dimensionality, followed by global average pooling to produce a channel-wise global mean vector *F*_gap_. This vector is then broadcast across the entire feature map and added to the local feature *F*_1_ thereby enabling the implicit propagation of global semantic information into local regions, as defined in Equation (10):

(10) $$\begin{array}{l} F_{gap\;}=\mathrm{GAP}({\mathrm{Conv}}_{1\times 1\;}(F_{1\;}))\\ F_{2}=F_{1}\;+F_{\mathrm{gap}}\;\cdot 1_{H\times W}\; \end{array}$$

The GPFN integrates the traditional GDFN module with an implicit global propagation mechanism. By computing the global mean and propagating it to each local position, GPFN significantly improves the coherence between local texture restoration and the global semantic structure.

### 3.3. Adaptive Content Guidance Module

The feature fusion mechanism within encoder–decoder architectures is crucial to the performance of image restoration tasks such as deraining, defogging, and desnowing. Conventional fusion methods (e.g., element-wise addition and adaptive blending) rely on the fusion weights to integrate features from different hierarchical levels. However, when deep-layer features correspond to local regions in shallow layers, direct fusion may lead to receptive field misalignment, which introduces spatial semantic inconsistencies and degrades the overall restoration quality. To mitigate this problem, an ACGM is proposed. This module primarily consists of channel-wise and spatial-wise attention components [4].

Traditional channel and spatial attention mechanisms are typically computed in isolation, resulting in limited interaction between them and reduced precision in multi-level feature fusion. This limitation becomes particularly pronounced in defogging tasks, where spatial attention often models haze distribution using a single-channel spatial weight map, thereby failing to capture semantic variations across channels, such as differences between texture and illumination. As a result, the spatial attention mechanism has difficulty accurately modeling channel-wise haze distribution. To address this issue, a coarse-to-fine strategy is proposed to dynamically generate a Spatial Importance Map (SIM) for each channel, enabling more fine-grained spatial adaptation. The ACGM utilizes the learned spatial weights to modulate feature responses, thereby facilitating the adaptive fusion of low-level encoder features with their corresponding high-level counterparts. The module consists of three stages: input fusion, feature processing, and feature output.

In the input fusion stage, low-level features from the encoder and the corresponding high-level features from the decoder are combined to perform initial fusion, and the resulting fused features are passed to the feature processing stage for modulation weight generation.

In the feature processing stage, spatial and channel attention mechanisms are employed to modulate the features and compute the corresponding attention weights, as illustrated in Figure 2. Let $X\in {\mathbb{R}}^{C\times H\times W}$ denote the input feature to this stage. The feature processing stage aims to generate a Spatial Importance Map (SIM) with the same channel and spatial dimensions as *X*. First, channel attention and spatial attention are computed separately. For channel attention $W_{c}\in {\mathbb{R}}^{C\times 1\times 1}$, channel attention coefficients are generated by applying two fully connected layers with nonlinear activations in sequence, thereby introducing nonlinear cross-channel interactions and enhancing the model’s ability to capture inter-channel dependencies. The resulting coefficients are then used for channel-wise recalibration. For spatial attention $W_{a}\in {\mathbb{R}}^{H\times W}$, a spatial importance map is computed by combining global average pooling (GAP_c_) and global max pooling (GMP_c_) along the channel dimension. GAP_c_ captures global feature distributions, while GMP_c_ highlights local saliency features, together enabling the adaptive representation of informative regions. By leveraging both spatial and channel attention to assign distinct importance to channels and spatial locations, the model’s defogging performance is further enhanced. This process is defined in Equation (11):

(11) $$\begin{array}{l} W_{c}=\mathrm{ReLU}\left(FC_{2}\left(FC_{1}\left(GAP(X)\right)\right)\right)\\ W_{s}=\sigma \left({\mathrm{Conv}}_{7\times 7}\left[{\mathrm{GAP}}_{c}(X),{\mathrm{GMP}}_{c}(X)\right]\right) \end{array}$$

Then, the channel attention weights *W*_c_ and spatial attention weights *W*_s_ are combined via element-wise addition to facilitate fine-grained interaction across spatial and channel dimensions, thereby improving the precision of attention weight assignment. This process is defined in Equation (12):

(12) $$W_{coa}=W_{c}+W_{s}$$

To obtain the final refined and channel-specific weight map *W*, each intermediate weight *W*_coa_ is further modulated according to its corresponding input feature, forming a content-guided fine-grained weight modulation mechanism. Each *W*_coa_ is rearranged alongside its corresponding input channel in *X* via a channel shuffle (CS) operation and further processed by a following convolutional layer, which helps reduce the overall number of learnable parameters. This process is defined in Equation (13).

(13) $$W=\sigma \left(G{\mathrm{Conv}}_{7\times 7}\left(\mathrm{CS}(\left[X,W_{\mathrm{coa}}\right])\right)\right)$$

Here, *σ*( ) denotes the Sigmoid activation, CS( ) denotes the channel shuffle operation, and GConv_7×7_ denotes a 7 × 7 grouped convolution. The grouped convolution ensures that each output channel depends exclusively on its corresponding input feature and weight. This design enforces strict alignment between the generated weight map and channel semantics, thereby effectively emphasizing informative features.

Finally, in the feature output stage, the fused features are first activated by a Sigmoid function and then passed through a 1 × 1 convolution layer to produce the final output feature *F_fuse_*. Additionally, a skip connection is employed to inject the original input feature into the final output, helping to alleviate gradient vanishing. This process is defined in Equation (14):

(14) $$F_{fuse}={\mathrm{Conv}}_{1\times 1}(F_{low}\cdot W+F_{high}\cdot (1-W)+F_{low}+F_{high})$$

where 1 denotes a matrix filled with ones.

### 3.4. Loss Function

Traditional pixel-wise reconstruction loss constrains the similarity between the restored image and the ground truth by evaluating differences at the individual pixel level. However, it essentially optimizes each pixel’s intensity independently, thereby ignoring the consistency of intensity relationships within local regions of the image. For instance, in hazy scenes, contrast relationships in the original image—such as brightness differences between foreground objects and the distant sky—are often attenuated by atmospheric scattering. In rain- and snow-degraded images, the random overlay of noise pixels disrupts local intensity ordering, such as brightness gradients along continuous wall textures. Relying solely on pixel-wise constraints may lead the model to generate reconstructions that are numerically smooth but perceptually unrealistic—for example, producing unnatural brightness transitions in sky regions after defogging. To better supervise the discrepancy between the generated and ground truth images during training, we employ a combination of reconstruction loss and correlation loss to optimize the network. The definitions of these loss functions are as follows.

The pixel-wise reconstruction loss quantifies the similarity between the final output and the ground truth image, enforcing pixel-level accuracy. This is formally defined in Equation (15):

(15) $$L_{rec}=∥I^{hq}-I^{gt}{∥}_{1}$$

Here, *I^hq^* denotes the restored high-quality image, and *I^gt^* represents the ground truth image.

The correlation loss $\left(L_{cor}\right)$ [24] employs the Pearson correlation coefficient *ρ* to measure the global linear correlation between the restored image *I^hq^* and the ground truth image *I^gt^*, as defined in Equation (16):

(16) $$\rho \left(I^{hq},I^{gt}\right)=\frac{\overset{3HW}{\underset{i=1}{\sum }}\left(I_{i}^{hq}-{\bar{I}}^{hq}\right)\left(I_{i}^{gt}-{\bar{I}}^{gt}\right)}{3HW\cdot \sigma \left(I^{hq}\right)\cdot \sigma \left(I^{gt}\right)}$$

The corresponding correlation loss is defined in Equation (17):

(17) $$L_{cor}=\frac{1}{2}\left(1-\rho \left(I^{hq},I^{gt}\right)\right)$$

When *ρ* = 1, the loss equals 0, indicating perfect positive correlation; when *ρ* = −1, the loss reaches its maximum value of 1, indicating perfect negative correlation. By minimizing $L_{cor}$, the model is optimized to maintain global intensity consistency between the restored and ground truth images.

(18) $$L=L_{rec}+\beta L_{cor}$$

where *β* is a weighting factor that balances the contribution of the correlation loss.

## 4. Experiments

### 4.1. Datasets and Experimental Settings

To ensure fair comparison with existing adverse-weather restoration studies, we use 9000 synthetic snowy images from Snow100K [25], 1069 real raindrop images from Raindrop, and 900 rain-fog composite images from Outdoor-Rain [26] as the training set. For evaluation, we employ Test1 [6], Raindrop [6], Snow100K-L, and Snow100K-S as the test sets [25]. In addition, 1329 real adverse-weather images from Snow100K-Real are used for generalization validation.

All experiments are conducted using PyTorch 2.3.0 on an NVIDIA RTX 4080 platform. The input image size is set to 128 × 128, the batch size is set to 3, and the model is trained for 300,000 iterations. The network adopts a four-stage progressive architecture with {L_1_ = 4, L_2_ = 4, L_3_ = 6, L_4_ = 8} residual blocks at each stage and a base channel number of C = 48. A multi-head progressive strategy is employed for the attention mechanism, with the number of heads set to {h_1_ = 1, h_2_ = 2, h_3_ = 4, h_4_ = 8} at each stage. The AdamW optimizer [27] is adopted (β1 = 0.9, β2 = 0.999), together with cosine annealing learning rate scheduling. The initial learning rate is set to 3 × 10^−4^, which is linearly warmed up for 92,000 iterations and then decayed to 1 × 10^−6^ following a cosine annealing schedule. Additionally, random horizontal and vertical flipping are applied as data augmentation strategies to further improve the generalization ability of the model.

### 4.2. Ablation Study

#### 4.2.1. Module Ablation

To validate the effectiveness of VMT and assess the contribution of each major component, we conduct ablation experiments on the Snow100K-S dataset. We adopt Peak Signal-to-Noise Ratio (PSNR) and Structural Similarity Index (SSIM) as two commonly used image quality metrics to evaluate the quality of the restored images and their similarity to the ground-truth images.

(1) DRSA: DRSA is introduced into the baseline model.
(2) DRSA + GPFN: Both DRSA and GPFN are incorporated into the baseline model.
(3) V-Mamba: V-Mamba is introduced into the baseline model.
(4) DRSA + GPFN + V-MAMBA: DRSA, GPFN, and V-Mamba are all introduced into the baseline model.

As shown in Table 1, owing to the complementary strengths of Mamba and Transformers, VMT is able to restore degraded details more effectively. Compared with the baseline model, VMT achieves improvements of 0.57 dB in PSNR and 0.67% in SSIM. Furthermore, the component-wise ablation results indicate that PSNR consistently exhibits stable gains, while the maximum difference in SSIM is only 0.0011, reflecting an SSIM saturation effect.

Since the overall image quality after restoration is already high, further improvements are primarily reflected at the pixel level, with the geometric structure remaining nearly unchanged, resulting in limited variation in SSIM. This substantially reduces the Mean Squared Error (MSE). As PSNR mainly reflects pixel-level errors, it shows a significant increase, while the structural similarity term is less affected, leading to only minor changes in SSIM.

#### 4.2.2. Ablation on Cross-Layer Feature Fusion Schemes

Skip connections integrate low-level features from the encoder with upsampled high-level features from the decoder, effectively combining local details with global semantics. Since high-level features contain richer global information, the fused features can more effectively guide the utilization of low-level information. The ACGM dynamically aggregates features between encoder and decoder layers via attention mechanisms, replacing the concatenation operation and the 1 × 1 convolution for channel adjustment commonly used in conventional U-Net architectures. Compared to traditional methods, ACGM adaptively selects and weights features, significantly enhancing the effectiveness of cross-layer feature fusion.To further investigate the impact of ACGM placement on network performance, we conduct ablation experiments on two different fusion strategies using the Snow100K-S dataset:

Strategy 1: Features from the downsampled low-level encoder VMT modules are fused with outputs from the high-level decoder VMT modules via ACGM.

Strategy 2: Features from the high-level encoder VMT modules are fused with upsampled outputs from the low-level decoder VMT modules via ACGM.

As shown in Table 2 Strategy 1 outperforms Strategy 2 by 0.24 dB in PSNR and 0.49% in SSIM. By downsampling high-level encoder features to match the resolution of low-level decoder features, Strategy 1 avoids information mismatch, improves the naturalness and quality of feature fusion, and effectively balances global semantics and local details, thereby enhancing the restoration capability of the model. Moreover, downsampling reduces the size of feature maps passed through skip connections, which lowers computational complexity. Resolution matching also enables the attention mechanism to more accurately select and weight features, further optimizing the fusion process. In summary, due to its superior performance in feature alignment, information balance, computational efficiency, and fusion effectiveness, Strategy 1 is adopted as the final model configuration in this work.

### 4.3. Comparative Experiments

#### 4.3.1. Quantitative Analysis

To comprehensively evaluate the performance of the proposed method, comparative experiments are conducted on both synthetic and real-world datasets, using two widely adopted image quality metrics, we compare it with several state-of-the-art approaches (1) Task-specific models: For snow scene restoration, five baselines including SPANet [28] and JSTASR [29] are selected; for rain and fog removal, five baselines such as CycleGAN [30] and Restormer [17] are used; and for raindrop removal, four baselines including pix2pix [31] and AttentiveGAN [32] are adopted. (2) Multiple degradation restoration models: Transformer-based models such as IDT [33] and NAFNet [34] are included. (3) Unified framework methods: We further compare with eight multi-task networks, including All-in-One [35], TransWeather [4], and Histoformer [14]. All comparison methods utilize the same datasets, training strategies, and inference procedures to ensure the fairness and comparability of the results.

As shown in Table 3. on synthetic datasets, VMT-Net achieves substantial improvements over the baseline model Histoformer across multiple benchmarks. For snow scene restoration, VMT-Net improves PSNR by 0.89 dB and 0.36 dB, and SSIM by 0.91% and 0.11%, respectively. For rain-fog and raindrop scenarios, VMT-Net performs on par with the baseline model. Compared to task-specific models such as DesnowNet (for snow removal) and RaindropAttn (for raindrop removal), VMT-Net still demonstrates a clear advantage.

The results indicate that VMT-Net markedly outperforms existing methods in snow-scene restoration. However, its advantages in rain–fog and raindrop scenarios are less consistent, suggesting varying adaptability to different degradation mechanisms: the model is more effective at handling large-area coverage and enforcing structural consistency (as in snow), whereas there is still room for improvement under strong local occlusions (e.g., raindrops, rain-fog). Overall, VMT-Net demonstrates clear advantages for adverse-weather image restoration, not only recovering fine details effectively but also preserving overall image quality.

#### 4.3.2. Qualitative Analysis

**Figure 4:**
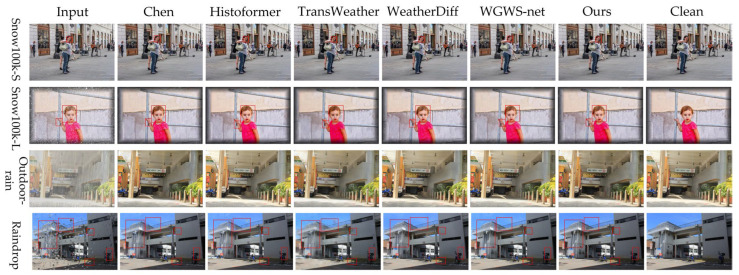
Visual comparison on snow, raindrop, and rain-fog restoration tasks.

To further validate the performance of VMT-Net, visual comparisons are conducted on the Test1, Raindrop, Snow100K-L, and Snow100K-S test sets, as illustrated in Figure 4. From the qualitative comparisons, we observe the following: in snow-degradation scenarios, our method effectively removes both small flakes and large accumulations, with particularly strong performance in restoring fine textures such as clothing. This improvement stems from the global–local complementarity of Mamba and Transformers, which facilitates reconstruction under large-area occlusions and texture recovery. However, in facial regions, the restoration of fine-grained textures (e.g., hair and skin) is not consistently stable, and artifacts may appear. Compared with methods such as WeatherDiff, our approach more accurately restores depth cues, although slight color darkening occurs under certain complex illumination conditions. In the raindrop removal task, the proposed method not only preserves edge sharpness but also better corrects optical distortions caused by raindrop refraction, outperforming existing approaches. VMT-Net achieves improved correction of geometric distortions induced by refraction while maintaining strong edge sharpness and structural consistency; nonetheless, small residual specular highlights remain for large, highly bright droplets.

Mechanistically, the channel–spatial recalibration in ACGM may amplify mismatched noise when emphasizing low-level texture channels, leading to localized color distortions; by contrast, the long-range dependencies captured by DRSA and V-Mamba enable the model to exploit unobstructed context to constrain reconstruction in occluded regions and improve structural consistency. When droplets are large and highly saturated, however, nonlinear refraction limits the available information, so detail and color recovery remain suboptimal.

**Figure 5:**
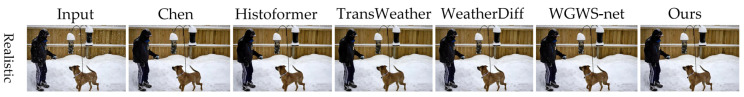
Visual comparison on real-world scenes.

In addition, we evaluated the generalization capability of the model in real-world scenarios and visually compared its performance with other state-of-the-art models, as shown Figure 5. In the real-snow test of the Snow100K dataset, VMT-Net removes snowflakes more thoroughly and delivers superior overall visual quality, fully demonstrating the architecture’s enhanced generalization to multi-scale degradation scenarios.

### 4.4. Application in Real-World Scenarios

**Figure 6:**
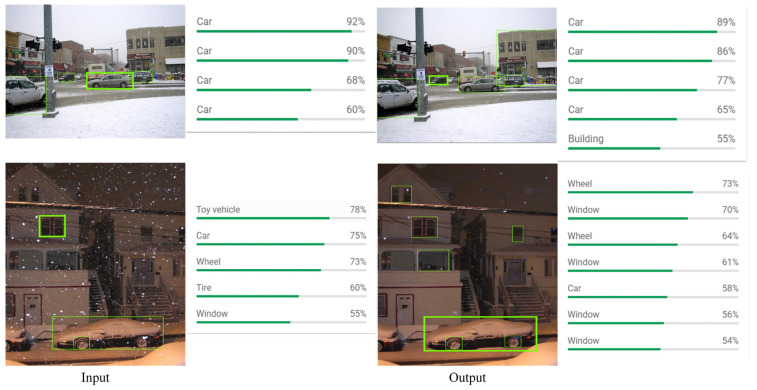
Restoration of two snowy scene images and their downstream detection results using Google API.

To further validate the practical applicability of the proposed method under adverse weather conditions and its potential benefits for downstream object detection tasks, Figure 6. presents a visual comparison of detection results before and after restoration. Due to occlusion from snowflakes, detection accuracy for “vehicles” and “buildings” drops significantly in the original images. Specifically, the detection accuracies for four vehicles are only 89%, 86%, 77%, and 65%, respectively, while the accuracy for “buildings” is just 55%.

After restoration, snowflake interference is effectively removed, and the detector’s recognition performance is significantly improved. The detection accuracies for “vehicles” increase to 92%, 90%, 68%, and 60%, respectively, and the accuracy for “buildings” is also greatly enhanced, resulting in a notable improvement in overall detection performance. Furthermore, with clearer images, the detector is able to accurately recognize additional objects such as “buildings” “windows” and “tires”.

These experimental results demonstrate that the restored images not only enable the detector to more accurately identify occluded or blurred targets, but also enhance the recognition of multiple object categories in complex scenes. Overall, the proposed method effectively mitigates the impact of adverse weather and improves recognition accuracy in downstream detection tasks.

## 5. Conclusions

We propose an adverse-weather image-restoration network that merges Transformer and Mamba architectures to balance between global semantic modeling and local-detail preservation. The network is built around a dual-branch VMT core: V-Mamba captures long-range dependencies, DRSA applies global self-attention, and a GPFN-based gated feed-forward path jointly models global and local contexts, thereby mitigating information loss and texture distortion. An ACGM assigns spatial weights to each channel, preventing semantic conflicts that arise from directly fusing deep and shallow features.

The experimental results show that VMT-Net achieves significant improvements over existing state-of-the-art models in two snow removal tasks, while maintaining comparable performance in raindrop and rain-fog removal tasks. However, VMT-Net still encounters certain challenges when handling complex weather scenarios, particularly in raindrop removal and strong light reflection scenes, where the restoration results occasionally exhibit incomplete detail recovery and residual highlights. Nevertheless, VMT-Net demonstrates strong adaptability, and future research could further refine its performance under extreme weather conditions to meet the requirements of real-time applications.

## Figures and Tables

**Table 1:** Quantitative comparison of ablation study results for VMT.

Method	PSNR	SSIM
Baseline	37.41	0.9656
DRSA	37.61	0.9717
DRSA + GPFN	37.73	0.9722
V-Mamba	37.64	0.9712
DRSA + GPFN + V-MAMBA	37.98	0.9723

**Table 2:** Quantitative comparison of ablation study results for ACGM.

ACGM	PSNR	SSIM
Not affiliated with ACGM	37.98	0.9723
Strategy 1	38.30	0.9747
Strategy 2	38.06	0.9698

**Table 3:** The quantitative comparison of three different weather restoration tasks.

	Image Desnowing					Deraining & Dehazing			Raindrop Removal	
	Snow100K-S		Snow100K-L			Outdoor-Rain			Raindrop	
	PSNR	SSIM	PSNR	SSIM		PSNR	SSIM		PSNR	SSIM
SPANet	29.92	0.826	23.7	0.793	CycleGAN	17.62	0.656	pix2pix	28.02	0.8547
JSTASR	31.4	0.9012	25.32	0.8076	pix2pix	19.09	0.71	DuRN	31.24	0.9259
RESCAN	31.51	0.9032	26.08	0.8108	HRGAN	21.56	0.855	RaindropAttn	31.44	0.9263
DesnowNet	32.33	0.95	27.17	0.8983	PCNet	26.19	0.9015	AttentiveGAN	31.59	0.917
DDMSNet	34.34	0.9445	28.85	0.8772	MPRNet	28.03	0.9192	IDT	31.87	0.9313
NAFNet	34.79	0.9497	30.06	0.9017	NAFNet	29.59	0.9027	MAXIM	31.87	0.9352
Restormer	36.01	0.9579	30.36	0.9068	Restormer	30.03	0.9215	Restormer	32.18	0.9408
All-in-One	–	–	28.33	0.882	All-in-One	24.71	0.898	All-in-One	31.12	0.9268
TransWeather	32.51	0.9341	29.31	0.8879	TransWeather	28.83	0.9	TransWeather	30.17	0.9157
Chen et al.	34.42	0.9469	30.22	0.9071	Chen et al.	29.27	0.9147	Chen et al.	31.81	0.9309
WGWSNet	34.31	0.946	30.16	0.9007	WGWSNet	29.32	0.9207	WGWSNet	32.38	0.9378
WeatherDiff64	35.83	0.9566	30.09	0.9041	WeatherDiff64	29.64	0.9312	WeatherDiff64	30.71	0.9312
WeatherDiff128	35.02	0.9516	29.58	0.8941	WeatherDiff128	29.72	0.9216	WeatherDiff128	29.66	0.9225
AWRCP	36.92	0.9652	31.92	0.9341	AWRCP	31.39	0.9329	AWRCP	31.93	0.9314
Histoformer	37.41	0.9656	32.16	0.9261	Histoformer	32.08	0.9389	Histoformer	33.06	0.9441
VMT-Net (ours)	38.30	0.9747	32.52	0.9272	VMT-Net (ours)	31.89	0.9336	VMT-Net (ours)	33.09	0.9441

Note: “–“ indicates that results are not available.

## Data Availability

The relevant code and data will be made available on GitHub at the following link: https://github.com/awcea/Adverse-Weather-Image-Restoration-Method-Based-on-VMT-Net.git (accessed on 19 October 2025).
